# Supplementary material for: Are carbon emissions trading and green financial instruments synergistic? -Comprehensive quantitative research based on content analysis
Source: PLoS One. 2024 Mar 7;19(3):e0298601. doi: 10.1371/journal.pone.0298601 (PMC10919601; doi:10.1371/journal.pone.0298601)
Supplement: S1 Data — (ZIP) [file pone.0298601.s001.zip › Data/地级市Z_F工作报告.zip!/╦─┤¿/╣π░▓╩╨/└┤╫╘╣1⁄2╓┌║┼í╛┬φ┐╦╩2╛▌═°í┐_2014.docx]

各位代表:

我代表市人民政府,向大会报告过去一年的政府工作,对今年工作提出建议,请各位代表审查,并请市政协委员提出意见。

2013年工作回顾

过去一年,是广安发展历程中克难攻坚卓有成效的一年。在省委、省政府和市委的坚强领导下,在市人大、市政协的监督支持下,市政府认真履行职能,团结依靠全市人民,坚持“科学发展、加快发展”的指导思想,大力实施“三大战略”,奋力推进“两个跨越”,广泛开展“感恩小平·我为小平家乡发展作贡献”主题活动,积极应对宏观经济下行压力加大、传统骨干产业结构性萎缩等不利影响,统筹稳增长、调结构、促改革、惠民生,全市经济社会实现了持续较快发展。

经济保持平稳较快增长。面对复杂严峻的宏观经济形势,我们科学研判形势变化,及时出台抓投资、强工业、促消费和加快推进新型城镇化等政策措施,投资、消费、出口保持快速增长势头,主要经济指标增速跃居全省前列,实现地区生产总值835.1亿元、比上年增长(下同)10.8%,地方公共财政收入38.6亿元、增长17.7%,全社会固定资产投资672.4亿元、增长28.3%,社会消费品零售总额292.1亿元、增长14.6%,外贸出口9.8亿美元、增长39.8%,增速分别居全省第6、第9、第3、第5、第4位。新的经济增长点加速培育,创新构建了“6+3+1”十点驱动发展格局,县域经济追赶跨越竞相发展;广安经开区、枣山园区、协兴园区建设如火如荼,开工建设和实施项目59个、总投资达827.7亿元。重大项目建设取得突破,160个重点项目建设完成投资238.3亿元、增长66.8%,玖源化工一期、鼎鑫铜铝型材等35个重大项目竣工投产,宏威聚酰亚胺新材料、鸿岱科技等70个项目开工建设,七一水库改扩建等55个项目快速推进;广安港开港试运营、配套工程推进扎实,巴广渝、遂广高速公路广安段加快建设,开行广安到成都动车前期工作有序推进。积极破解要素制约,成功争取中省资金127亿元、用地指标2万余亩,分别增长10.4%、121.8%;银行业金融机构新增信贷83.3亿元、增速居全省第6位,直接融资77.2亿元。

现代产业体系加速构建。加快推进新型工业化进程,规上工业增加值增长11.5%,在全省排位比2012年上升5位;工业对GDP的贡献率达49.7%。新培育规上企业90户,新增亿元企业67户,总数分别达494户、307户。信息技术、新能源、新材料、高端装备、节能环保等战略性新兴产业和医药化工、有色金属等潜力产业加快发展,实现产值170亿元,占工业总产值比重上升2.3个百分点。广安经开区纳入全省千亿园区培育计划并创建为全国循环化改造试点示范园区,邻水工业园区纳入省500亿园区培育计划,岳池城南工业园、武胜街子工业园被评为四川省小企业创业示范基地。加快推进现代农业建设,成功创建为国家现代农业示范区,“产村相融”提质扩面,“千斤粮、万元钱”粮经复合型产业基地规模拓展,粮食总产实现“七连增”,农民现金收入连续5年高位增长;新型农业经营主体不断壮大,新增规模以上农业产业化龙头企业10户、农民合作组织162个,联结带动农户1.1万余户;申报认证绿色农产品14个、有机农产品6个,欧阳晓玲蜜梨获中国驰名商标,广安蜜梨、邻水脐橙被认定为国家地理标志保护产品;农业机械化综合水平达到49%。加快推进现代服务业发展,实现服务业增加值247.7亿元、增长10.5%;广安成为省级服务业综合改革试点市和现代物流试点示范市,天津亿联、临港置业等大型物流企业相继入驻,川渝建材家居、泰合国际商贸城等19个重大物流商贸项目快速推进;红色文化影视城等景区加快建设,宝箴塞、神龙山巴人石头城、华蓥山天意谷创建国家4A级旅游景区通过省检,邓小平故里旅游景区成功创建为国家5A级旅游景区,全市实现旅游收入135.3亿元、增长30.6%。

城乡统筹发展步伐加快。加快推进以人为核心的新型城镇化建设,全域推进城乡建设规划编制工作,开展城市总规修编及各园区控详规划编制;城镇建设快速推进,市政道路加快建设,园区骨架路网初具雏形,县城规模不断扩张,5个省级重点小城镇建设启动实施,全市城乡建设完成投资126.9亿元、增长28.2%,新增城镇面积16.2平方公里,城镇化率达34.3%;城市管理全面加强,获中国智慧城市推动奖;房地产业健康发展,启动试点新型住宅产业,超额完成省定保障性住房建设任务。加快推进新农村建设,新(改、扩)建农民新村462个,建设新农村综合体5个,204个渠江流域灾后重建新村全部竣工。统筹推进城乡交通建设,开工建设环城公路东南段、广岳大道、枣彭路,广华快速通道、港前大道前期工作加快推进,新建农村公路1222公里,新增和改造农村客运线路41条。统筹实施农业综合开发项目,建设农田水利基本建设大示范区12万亩;完成病险水库除险加固125座,新增有效灌面8.5万亩;新建供水工程133处,解决了27.9万人饮水安全问题。

改革开放持续推进。推进行政区划调整,完成前锋新区筹建工作。改革广安经开区、枣山园区、协兴园区财政、人事和行政体制,充分激发园区自我发展活力。食品药品安全监管体制改革基本完成,事业单位分类改革、“营改增”试点、重要资源性产品价格改革及教育、卫生改革稳步推进,公务卡结算制度改革全面实施。创新投融资体制机制,推进政府投资项目管理改革,出台重大项目建设实施、BT和BOT管理、抽签招标确定中标人、工程变更管理、中介服务管理、消极作为问责等一批管理制度;争取人行成都分行、省银监局出台支持广安发展政策;启动邓小平故里发展基金筹建工作。深化行政审批制度改革,调整取消行政审批项目43项,取消行政事业性收费8项;加快公共资源交易改革,政务服务中心标准化建设深入推进。切实落实民营经济发展政策措施,大力促进非公经济快速发展;整合重组市属国有企业。创新区域合作体制机制,国家西部承接产业转移示范区获国家发改委批复,成功争取省政府出台广安川渝合作示范区建设支持政策,国家支持示范区建设的179个项目到位资金30.2亿元。组织参加全国知名民企入川、央企入川、港澳企业四川行等重大招商活动,签约引进亿元以上投资项目578个,到位资金702.1亿元、增长27.2%,中国建材、中国医药、正威国际、富盈集团等一批央企和知名民企相继投资广安。大力实施创新驱动发展战略,与中关村科技园区建立战略合作关系,搭建创新驱动发展新平台。引进实施重大科技成果转化项目18个。培育国家级高新技术企业8家、省级创新型企业12家,新增省级企业技术中心4家,申请商标注册1830件、专利640件。省级可持续发展实验区建设稳步推进,获全国科技进步工作先进市。

民生社会事业协调发展。全年民生支出114.3亿元,占公共财政支出的70.1%。城镇居民人均可支配收入22210元,农民人均纯收入8492元,分别增长11.2%、13.6%。惠民工程深入实施,筹集资金63.5亿元,完成省市民生项目138个。“两馆”建设加快推进,邓小平缅怀馆完成土建工程,邓小平故居陈列馆完成改陈设计及评审。部省共建教育改革发展试验区成果丰硕,素质教育深入实施,义务教育均衡发展加快推进,学前三年毛入园率达77.1%,高考录取率创近十年新高,广安职业技术学院被确定为省级示范性高职院校立项建设单位。市文化中心、广安大剧院等重大文化项目加快实施,广播电视“村村通”等重点文化惠民工程稳步推进,新增全国重点文物保护单位2处。完成20个乡镇文体广场建设,获得省级以上体育锦标赛金牌18枚。创建全国文明城市成效显著,在同类提名资格城市中文明指数测评获第3名、未成年人思想道德建设测评获第4名。全面取消县级公立医院药品加成,启动实施大病保险试点,新型农村合作医疗补偿金达12亿元,传染病发病率比上年下降23.3%。稳定低生育水平,发放计生奖扶资金5936万元。支持大中专毕业生、返乡农民工、城镇就业困难人员就业创业,城镇新增就业5.12万人。统筹城乡社会养老保险制度全面实施,工伤、生育、补充医疗保险实现市级统筹,社会保险参保累计达269.9万人;完善低保标准与物价上涨联动机制,城乡低保标准分别提高10.3%、23%;城乡医疗救助35.2万人次,“五保”分散、集中供养标准分别提高73%、160%。加大扶贫开发力度,12.6万贫困人口脱贫。深入开展依法治市和“平安广安”建设,全省首批法治城市创建工作通过专家评审,武胜县创建为全国平安建设先进县;加强和创新社会管理,扎实推进城乡网格化服务管理,全面深化“五大民生警务”战略,深入开展防灾救灾大演练,有序推进基层单位应急管理规范化建设,启动矛盾纠纷大调解国家级服务综合标准化试点,及时处理群众信访问题,积极防范和化解社会矛盾,人民群众安全感进一步提升;依法严厉打击各类刑事犯罪,“黄赌毒”得到有效遏制。全面深化安全生产隐患大排查、大整治,安全生产形势保持稳定。加强食品药品安全监管,未发生重大食品药品安全事故。民族、宗教政策全面落实。国防动员、双拥共建、民兵预备役和人民防空工作进一步加强。

生态建设和环境保护成效明显。综合治理城乡生态环境,加强天然林保护,巩固退耕还林成果59.9万亩,完成营造林53.3万亩,综合治理岩溶地区50平方公里,实施地质灾害和矿山环境恢复治理,森林覆盖率达到38%;启动实施最严格的水资源管理制度,加强西溪河流域水环境综合治理,着力重建全民水库生态系统,开工建设广安垃圾发电厂,建成乡镇污水处理站40座,46个省级生态乡镇通过核查,279个行政村被命名为“市级生态村”;加强淘汰落后产能和节能减排工作,关闭小煤矿27户,单位生产总值能耗下降4.4%,主要污染物排放控制目标全面完成。广安成功创建为国家森林城市,列入全国资源型城市可持续发展规划和国家循环经济示范城市创建城市。

政府自身建设不断加强。一年来,我们着力建设法治政府、服务型政府。主动接受人大及其常委会法律监督、工作监督和政协民主监督,办理人大代表议案、建议153件,办理政协委员提案247件,办复率和满意率均为100%。建立市政府常务会议会前学法制度,修订完善《政府工作规则》,完善市政府常务会议制度和办文、办会制度,促进政府工作规范高效运行。建立健全一系列经济管理制度,努力用制度规范和监督各项管理工作。积极探索人民群众参与政府决策、监督政府行政的新途径、新方法,启动实施“网络理政”,建设行政权力依法规范公开运行平台,推行重大事项“开放式决策”,适时举行政府新闻发布。严格执行改进作风各项规定,加强机关行政效能建设,75个单位、202人因效能问题被追究责任;从严查纠“四风”问题,加大审计监督力度,认真开展财政评审,支持查处违纪违法案件,政府形象进一步提升。

同时,统计服务、国资管理、粮食储备、住房公积金管理、信息化建设、供销、气象、外事、侨务、港澳台事务、档案、保密、地方志、防震减灾、老龄事业、邮政、保险、盐政、烟草专卖等工作取得新成绩,石油、天然气、电力、供水、通信等保障工作取得新进展,工会、共青团、妇联、科协、残联、贸促会、文联、社科联、红十字会、关工委以及各民主党派、工商联等为经济社会发展做出新贡献。

各位代表,过去一年,我们坚持科学发展、加快发展,通过综合施策保持了经济持续快速增长,主要经济指标增速快于全国全省;坚持解放思想、改革创新,通过创新体制机制构建了多点驱动发展格局,通过深化改革开放激发了各类市场主体活力;坚持以人为本、民生优先,把保障和改善民生作为政府的最大责任,通过统筹协调发展提升社会民生保障水平,尽力解决群众关心的民生难题。过去一年的成绩来之不易。这是省委、省政府坚强领导、关心支持的结果,是市委统揽全局、科学决策的结果,是全市人民克难攻坚、共同奋斗的结果。在此,我代表市人民政府,向全市干部群众,向人大代表、政协委员,向各民主党派、工商联、人民团体和各界人士,向驻广部队、武警官兵和公安民警,向所有支持广安改革发展的同志们、朋友们,表示衷心的感谢!

在总结过去一年工作的同时,我们也清醒地看到,全市经济社会发展和具体工作中还存在不少困难和问题:经济长期积累的结构性矛盾仍然突出,宏观环境不利因素和发展水平较低等客观因素叠加,一定程度制约着后续发展。特别是工业中传统资源型产业比重过高,相当部分企业和产品竞争力不强;现代农业基础薄弱,服务业比重偏低,经济向好发展的基础还不牢固。基本公共服务与群众期盼还有较大差距,保障和改善民生的任务仍然繁重。政府公共管理和依法治理能力还需提升,政风行风还需进一步改进,少数干部还存在不务实、不廉洁甚至腐败等问题。我们要直面这些困难和问题,在今后的工作中认真研究并加以解决。

2014年主要目标任务

2014年是邓小平同志诞辰110周年,是广安在新的起点上全面深化改革的开局之年。今年政府工作的总体要求是:深入贯彻党的十八大、十八届三中全会、省委十届三次、四次全会精神,认真落实市委四届六次全会和市委经济工作暨城镇化工作会议部署,以改革统领经济社会发展全局,坚持“科学发展、加快发展”不动摇,深入实施“三大战略”,精心筹办邓小平同志诞辰110周年纪念活动,保持经济发展定力,挖掘经济增长潜力,释放改革创新活力,着力保障和改善民生,继续保持高于全国全省的发展速度,推动广安经济社会持续快速健康发展。

今年全市经济社会发展的主要预期目标是:地区生产总值增长11%左右,全社会固定资产投资增长25%,社会消费品零售总额增长13.5%,地方公共财政收入增长15%,城镇居民人均可支配收入增长11.5%,农民人均纯收入增长13.5%,人口自然增长率控制在5.6‰以内,完成省下达的节能减排任务。

研究提出今年经济社会发展预期目标,我们充分考虑了以下因素。一方面,广安可望继续保持较快发展的良好势头。中央确立了稳中求进、改革创新的工作基调,继续实行积极的财政政策和稳健的货币政策,强调保持宏观经济政策的连续性和稳定性,并出台了一系列激发经济增长活力的重大改革政策,这为广安加快发展创造了有利的外部条件。随着小平同志110周年诞辰的日益临近,中省对广安的支持力度更大,海内外对广安的关注度更高,社会各界对小平同志的感恩之情将进一步转化为广安发展的现实驱动力。近几年来我市在保持经济合理增长的同时,大力推动经济结构调整,积极发展新兴产业,改造提升传统产业,转型升级发展已有了良好的开端;加之一大批新开工项目加快形成实物工程量并逐步释放产能,新型城镇化快速推进,综合交通枢纽加速构建,这为广安蓄势发展提供了有力支撑。同时,我市拥有川渝合作示范区、国家西部承接产业转移示范区、国家级经开区、国家现代农业示范区、国家商标战略实施示范城市等发展平台和优惠政策,必将持续吸引更多的市场要素集聚,释放出巨大的政策红利。另一方面,广安必须加快发展、奋力追赶跨越。对广安来讲,“人口多、底子薄、欠发达”的基本市情和“发展不够、发展水平不高”的主要矛盾还没有改变。省委十届三次全会后,各市州竞相跨越的势头十分强劲,2013年生产总值上千亿的市又新增了3个,全省已有12个市州跨进了千亿行列。广安要实现市委四届六次全会确定的迈入全省经济发展第一方阵、与全国全省同步全面建成小康社会的目标,任重而道远,必须负重奋进、加速赶超。同时,我们也要顺应经济转型发展的大趋势。当前,我国经济长期向好的基本面没有变,但发展已全面进入经济增长换挡期、结构调整阵痛期和前期刺激政策消化期,经济形势严峻复杂,经济下行压力仍然较大。由于我市经济结构性矛盾突出,各方面要素供给和资源环境约束趋紧,我们在确定经济增长目标时,应当为转方式调结构预留空间。

综合以上分析,我们确定了稳中求进、加压驱动的发展目标。客观地讲,这样的目标仍然是一个较高的目标,是需要艰苦努力才能够实现的目标。我们必须进一步增强责任意识、机遇意识、进取意识,在认清大势中加快发展,在提质增量中转型发展,在改革创新中跨越发展,在统筹兼顾中协调发展,以焕然一新的面貌迎接邓小平同志诞辰110周年。

2014年重点工作

做好今年政府工作,要以深化改革为强大动力,以调整结构为主攻方向,以改善民生为根本目的,统筹兼顾,突出重点,务求实效。

一、隆重热烈简朴务实办好纪念活动

把筹办好纪念活动作为今年最首要的任务,以纪念活动总揽各项工作,推动经济加快发展、民生持续改善。

精心开展纪念活动。按照中省安排,办好邓小平同志诞辰110周年相关纪念活动,协助举办“四川省纪念邓小平同志诞辰110周年座谈会”、邓小平缅怀馆开馆仪式和“深入学习邓小平改革开放思想,全面深化改革,实现中华民族伟大复兴”理论研讨会,配合中国文联做好“送欢乐 下基层”文化惠民演出、中国音协做好春天的故事——纪念邓小平同志诞辰110周年歌曲征集活动,组织开展《春天的故事》大型群众文艺汇演活动,努力增强纪念活动感染力、影响力。

抓好纪念设施及惠民项目。全力推进主题纪念设施建设,确保5月19日前邓小平故居陈列馆完成改陈并对外开放、6月底前邓小平缅怀馆投入试运行。切实抓好市民广场、小康示范村等建设,加快实施西溪河生态堤防、协兴历史文化街区等惠民工程,全力推进枣彭路和邓小平故里景区道路等基础设施建设。加快实施感恩作贡献项目,确保按期完成建设任务。

全力争取各方支持。把握中省政策取向和资金投向,精心策划包装项目,加大衔接汇报力度,努力在重大政策、重大项目、中省资金争取和落实上实现突破,力争项目资金比去年增加20%以上。深化“感恩小平·共建广安”活动,全方位、多层面争取各级部门、商会、企业及海内外各界人士捐资捐建、投资兴业,聚集各方力量支持小平家乡建设与发展。

二、推动重要领域改革取得新突破

坚持把改革贯穿于经济社会发展各领域各环节,以经济体制改革为重点,区别情况,分类推进,抓好牵一发而动全身的举措,力求取得实质性进展,更多释放改革红利。

深化行政体制改革。着力优化投资建设项目和生产经营活动行政审批,依法取消和简化前置性审批,全面公布行政审批权力清单,清理非行政许可审批项目,真正实现让市场决定资源配置。基本完成市县政府机构改革,继续推进事业单位改革,巩固深化园区管理体制改革。实施工商登记制度改革,落实认缴登记制,由先证后照改为先照后证,由企业年检制度改为年报公示制度。

深化财税体制改革。实施全面规范、公开透明的预算制度。着力把所有政府性收入纳入预算,实行全口径预算管理。各级政府预算和决算都要向社会公开,部门预算要逐步公开到基本支出和项目支出,所有财政拨款的“三公”经费都要公开,打造阳光财政,让群众看明白、能监督。推进分税制财政体制改革,合理确定市与三个园区的收入分享比例,合理划分市与区(市、县)事权范围,理顺政府间收入分配关系。全面落实中省税收制度改革的各项部署,做好“营改增”试点扩面工作。建立综合治税平台,加强税源监控,确保应收尽收。深化财政专项资金管理改革。强化财政支出绩效评价。建立规范的地方政府举债融资机制,把地方政府性债务纳入预算管理,推行政府综合财务报告制度,防范和化解债务风险。

深化金融体制改革。健全现代金融体系,推进县级农村信用联社改制为农村商业银行,鼓励民间资本发起设立中小型银行等地方性金融机构和新兴金融组织。创新金融信贷方式,拓宽金融产品服务,扩大信贷投放规模,加大实体经济和中小微企业信贷支持力度。深化投融资体制改革,做强做实政府性投融资平台,支持民间资本进入公共服务领域。充分发挥邓小平故里发展基金作用,强化多层次资本市场培育,推进辖区内企业通过多层次资本市场融资。强化金融、财政、产业政策协同作用,提升金融服务实体经济能力。着力防范化解各类金融风险,营造良好金融生态环境。

增强各类所有制经济活力。坚持和完善基本经济制度,加快发展混合所有制经济,建立健全现代企业制度和公司法人治理结构。完善国有资产管理体制,改革国有资本授权经营体制,完善国有资本经营预算制度,提高国有资本收益上缴公共财政比例。鼓励支持非公有制经济发展,消除非公有制企业进入特许经营领域障碍,在更多领域放开竞争性业务,为民营经济发展提供更加广阔的空间。

三、开创高水平对外开放新局面

着力构建开放型经济新体制,推动全方位对外开放,倒逼深层次改革和结构调整,加快培育经济发展新优势。

加快推进川渝合作示范区和承接产业转移示范区建设。跟踪落实6大类19项支持政策和项目,进一步抓好政策和项目落地;全面对接渝广两地产业、资本、市场、人才及公共服务,加快推进产业协作配套、交通互联互通、生态环境共建共治、公共服务对接共享;主动接受成都首位城市辐射,积极深化与长三角、珠三角等发达地区的合作,加强与北京海淀区的联系,全面提升区域合作、承接产业转移的质量和水平。

不断提升招商引资规模和实效。创新招商引资平台和载体,拓展招商引资新路径,积极参加知名企业四川行、西博会、渝洽会等展会活动,组织开展中外知名企业广安行等投资促进活动,着力优化引资结构,突出招大引强、招群引链,完善招商引资考核办法,推动项目落地建设,提高招商引资履约率和资金到位率,力争招商引资到位资金800亿元。

大力发展外向型经济。积极开拓国际市场,努力扩大对外贸易份额,力争实现外贸进出口总额10.2亿美元,其中出口10亿美元。调整优化对外贸易结构,重点支持生产型出口企业发展,培育5000万美元以上生产型企业1户、3000万美元以上生产型企业2户。提升外资利用水平,实际利用外资4000万美元以上。

四、深入实施多点多极支撑发展战略

强化多点多极支撑,加快县域经济发展和产业园区建设,加速培育新的经济增长点,保持各地竞相发展的良好态势。

加快推进三大园区建设。坚持产城一体、错位发展,把三大园区建成主城区的产业新城和市域经济的重要增长极。广安经开区重点是高起点规划建设奎阁、新桥片区,加快滨江新城拓展,积极发展总部经济,突出发展精细化工、有色金属等主导产业和新兴产业,加快建设千亿级产业园区。枣山园区重点是推进站前广场、重点物流通道及配套设施建设,加快实施粮食、建材、汽车等重大物流项目,规划建设枣山总部经济群,着力建设对接重庆、辐射川东北的商贸物流集散地。协兴园区重点是推进重大文化和旅游项目加快建设,全面建成酒店集群,完善提升旅游要素,加快打造国家重点文化产业集聚区和世界旅游目的地。

大力发展县域经济。依托区市县园区发展平台,培育各具特色、各具优势的县域支柱产业,实现经济总量、财政收入和群众收入快速增长,不断夯实底部基础。加快建设高滩、罗渡、街子、溪口等新型工业园区和前锋商贸物流园区、华蓥山旅游度假区,推进广安临港都市产业园、渝广共建邻水机电产业园、华蓥电子信息产业园、武胜节能环保新材料工业园建设,支持岳池建设中国西部医药产业城,形成一批支撑县域经济跨越发展新的增长点。加强城乡统筹,因地制宜发展壮大一批工业镇、商贸镇、旅游镇,努力形成县域经济次中心。充分发挥民营经济在推动县域经济发展中的重要作用,认真落实财税、金融、用地等支持政策,激发民营经济发展活力和创造力,不断壮大县域经济实力。

五、增强投资和消费拉动经济的主引擎作用

充分发挥投资的关键作用、消费的基础作用,努力扩大投资规模和消费需求,持续推动经济较快增长。

优化投向扩大投资。加快投融资体制改革,推进投资主体多元化。主动对接国家政策支持方向和投资结构调整方向,充分用好中省项目资金,发挥投融资平台作用,突出抓好基础设施、民生保障、节能环保等领域的投资,促进政府性投资持续增长。加大工业、服务业等产业发展投资力度,支持企业加大技术改造和创新投入,培育、引进一批投资体量大、综合效益好、带动能力强的先进制造业和现代服务业项目。进一步放开投资领域,降低准入门槛,优化投资环境,引导更多的民间资本投入到经济建设和民生事业。重点抓好393个投资1000万元以上的重大项目,其中开工建设纳米OGS触摸屏、利尔精细化工、广安国际会展中心等144个项目;加快推进玖源聚苯硫醚新材料、渠江水淹区官盛移民安置房建设等159个项目;全面竣工诚信化工二期、鼎恒锂电池一期、环城公路东南片区段等90个项目,确保全社会固定资产投资完成840亿元以上。

努力增加有效消费需求。深化流通体制改革,降低流通成本,促进物流配送、快递业和网络购物发展,充分释放消费潜力。打造中央商务区、城市商圈、特色街区,推进家居建材、汽车销售等专业市场建设,形成服务业聚集区,聚集人气,提升商气。加快建设农产品交易市场、商贸重镇、社区服务店,加强产销和农超对接,推进农村商业连锁经营和统一配送,进一步完善农村市场流通体系。推进餐饮住宿等传统消费升级,积极培育信息消费、健康养老、电子商务、旅游文化等消费热点,创新服务消费、信贷消费、租赁消费等新型消费业态。积极搭建各类促销平台,努力争取国家级、省级重大节会活动在广安召开,不断繁荣会展和节假日消费市场。

六、以创新支撑和引领产业转型升级发展

牢固树立转型才能更好发展的理念,深入实施创新驱动发展战略,加快转方式调结构,不断提升经济发展质量和效益。

加强创新能力建设。加快科技体制改革,促进科技与经济社会发展紧密结合。继续探索借脑借智发展新路径,借助国内外知名学者、企业领军人物加强技术支持、投资促进和产业发展指导。深化与中关村、中国农科院的务实合作,实施重大科技成果转化项目15个以上。发挥企业主体作用,提升企业自主创新能力,新培育省级创新型企业15家、国家级高新技术企业8家。加快建设中小企业孵化中心,孵化科技型中小微企业15家以上。支持邻水经开区创建省级高新技术园区、广安区创建全国可持续发展实验区。加快推进信息化建设,不断提升信息化水平。

推进工业转型升级。大力培育新能源、新材料、节能环保、电子信息等新兴产业,加速壮大装备制造、医药、化工、有色金属等优势产业,力争新兴产业和潜力产业实现总产值250亿元,增长47%以上。改造升级传统产业,依法关闭、兼并重组地方小煤矿,支持水泥企业优化整合,实施重大技改项目175个。实施大企业大集团培育计划,新培育销售收入10亿元以上企业3户,新增规上企业80户,规上工业增加值增长12%以上

加快现代服务业发展。扎实推进省级服务业综合改革试点市和现代物流试点示范市建设,大力发展生产性、生活性和新兴服务业,推动服务业发展提速、比重提升、水平提高,确保服务业增加值增长10%。加快建设旅游经济强市,精心筹办第4个中国旅游日广安主会场活动,启动华蓥山旅游整体开发,推进龙女湖国际旅游度假区等项目建设,广安红色文化影视旅游城形成初步接待能力,促进文化与旅游产业融合发展。推进枣山、临港、前锋、万善、高兴、渝邻等物流园区建设,打造物流集聚区。大力发展商贸流通业,新增规模以上服务业企业32户。着力发展电子商务、文化创意、服务外包等新兴服务业。加快发展金融业,进一步完善金融体系,支持广安经开区、广安发展建设集团发行债券,正式营运邓小平故里发展基金,力争引进全国性股份制银行1家,全市新增信贷100亿元、直接融资100亿元以上、保险覆盖面扩大10%。健康发展房地产业,推进新型住宅产业化。

七、促进农业现代化和农村改革发展

坚持把解决“三农”问题作为工作的重中之重,全面激发农村改革发展活力,有效促进农业增效农民增收。

大力发展现代农业。狠抓粮食生产,稳定播种面积,推广良种良法,确保粮食稳产增产。深入推进农业结构调整,围绕主导产业和特色优势产业,实施“千斤粮、万元钱”工程,加快现代农业、现代林业、现代畜牧业产业基地建设。扎实推进标准化生产,大力培育农产品品牌,加强农产品质量监管,积极发展绿色农产品、有机农产品。发展壮大加工型、流通型龙头企业,不断提升农业产业化经营水平。切实加强以水利为重点的农业基础设施建设,抓好山水田林路综合治理,开工建设回龙寺、猫儿沟、龙滩等中型水库。

多渠道促进农民增收。全面落实强农惠农富农政策,从多方面帮助农民提高收入水平。充分挖掘种养业助农增收潜力,大力发展农产品加工业和乡村旅游业。巩固提升劳务转移输出数量和质量,抓好就地就近转移就业。加快建设农村产权交易市场,探索推进农民住房财产权抵押、担保和转让,保障农民公平分享土地增值收益。

积极推进农村改革。坚持和完善农村基本经营制度,赋予农民更多财产权利。保持农村土地承包关系长久不变,抓紧土地承包经营权及农村集体建设用地使用权确权登记颁证工作,引导承包地经营权有序流转,慎重稳妥进行农村土地制度改革试点。坚持家庭经营基础性地位,培育专业大户、家庭农场、农民合作社、农业企业等新型农业经营主体,发展多种形式适度规模经营。完善集体林权制度改革。健全农业社会化服务体系。切实尊重农民意愿,坚决维护农民合法权益。

加快建设幸福美丽新村。坚持“产村相融”路径,按照“业兴、家富、人和、村美”要求,积极筹措和整合各类涉农资金,统筹推进基础设施、新型村庄、产业发展、公共服务和社会管理综合配套,积极开展新型房屋试点示范,加快建设农业强、农村美、农民富的幸福美丽新村。全年新改扩建农民新村457个、新民居4.3万户。

八、推进以人为核心的新型城镇化

健全城乡发展一体化体制机制,坚持走以人为本、四化同步、优化布局、生态文明、传承文化的新型城镇化道路,遵循发展规律,积极稳妥推进,着力提升质量。

加强城镇化管理创新和机制建设。以国家新型城镇化规划为指导,完成组团城市总体规划、区市县总规修编、园区控详规划编制工作,做好相关规划的统筹衔接。继续优化城镇布局和形态,促进县城和小城镇协调发展。加强城市、小城镇和村庄规划管理,从严查处规划违法违规行为。探索建立多元化城镇建设投融资机制。提高城镇建设用地效率,优先发展公共交通,注重保护历史文化和自然景观,让城镇各具特色、宜业宜居,更加充满活力。

加快城镇基础建设。推进东南片区、广安经开区、枣山园区、协兴园区和官盛新区联动发展,完成环城路东南片区段、枣彭路建设,加快港前大道、官盛大桥建设和广前路改造,推进城北及西溪峡片区棚户区改造,扩大主城区发展规模,提升主城区承载能力;开行广安至成都动车;加快绕城高速前期工作,开工建设广华快速通道,建成广岳大道,加快构建组团城市交通网络体系,促进前锋、华蓥、岳池与主城区融合发展,推进邻水、武胜县城扩容提质,建设现代中等城市;加快集镇建设步伐,做强城市发展梯级支撑,全市新增城镇面积18平方公里,城镇化率提高3.2个百分点。

推进农业转移人口市民化。加快户籍制度改革,放开落户限制,解决好进城人口身份问题。通过发展城市产业提供更多市场化就业岗位,解决好进城人口就业问题。加快建设幸福美好家园,做好征地拆迁安置房、保障性住房建设和棚户区改造工作,开工建设保障性安居工程40596套,将新建公租房的30%房源向农民工供应,努力解决进城人口居住问题。推进城镇基本公共服务常住人口全覆盖,统一农民工和城镇职工参加失业保险管理,解决好进城人口市民待遇问题。

全力创建全国文明城市。围绕精神文明建设总要求,扎实抓好创建工作,力争成功创建全国文明城市。完成主城区主干道环境综合整治和市民广场景观工程,加强城市公园及便民设施建设,提升城市品质。完善物业管理,规范设置防护窗栏,整治乱停乱放,疏导流动摊贩,治理扬尘污染,提升城市管理水平。加快4G网络和下一代广播电视网络(NGB)建设应用,推进智慧城市、数字城市建设。积极推进社会志愿服务工作。

九、全面发展民生社会事业

持续加大民生投入力度,加快推进社会文化事业发展,解决好人民群众最关心最直接最现实的问题。

促进教育事业优先发展、公平发展。深入推进广安市教育改革发展试验区建设,新建幼儿园24所、城镇学校5所,改造农村薄弱学校100所,抓好友谊中学高中部迁建工作;推进城乡义务教育均衡发展,深入实施素质教育,加强师德师风建设,强化学校德育、艺体工作,建成市示范性综合实践基地;大力发展职业教育,深化产教融合、校企合作,培育高素质技能型人才,支持广安职业技术学院建设省级示范性高职院校,力争广安技师学院挂牌招生;支持民办教育健康发展。

推动医改向纵深发展。巩固全民基本医保,通过改革整合城乡居民基本医疗保险制度。完善政府、单位和个人合理分担的基本医疗保险筹资机制。加强城乡医疗救助、疾病应急救助。推进县级公立医院综合改革。创新社会资本办医机制。巩固完善基本药物制度和基层医疗卫生机构运行新机制。加强城乡基本公共卫生服务体系建设,启动建设市人民医院协兴分院,迁建市妇幼保健院,加快建设市全科医生培训基地,建设标准化乡镇卫生院15个、村卫生室60个;完善重大疾病防控体系和突发公共卫生事件应急机制,有效控制传染性疾病的流行和传播。坚持计划生育基本国策不动摇,落实一方是独生子女的夫妇可生育两个孩子政策

推进文化事业和文化产业发展。继续深化文化体制改革,完善文化经济政策,增强文化整体实力和竞争力。大力实施文化惠民工程,开工建设市博物馆,加快建设市文广新综合体等重点文化设施,推进广播电视“村村通”和乡村(社区)文化院坝建设;积极发展文化产业,不断净化文化市场,加强文化资源保护利用。扎实开展群众性体育活动,积极参加省“十二运会”。

统筹做好保障和改善民生工作。大力实施就业创业工程,帮扶高校毕业生等重点群体就业,城镇新增就业3.4万人,城镇登记失业率控制在4%以内。深化收入分配体制改革,努力缩小收入差距。完善统筹城乡社会养老保险制度,推进新型农村社会养老保险和城镇居民社会养老保险整合,实施基本医疗保险市级统筹,全面推进城乡居民大病医疗保险工作。推进社会救助制度改革,继续提高城乡低保水平,全面实施临时救助制度,强化孤儿保障、五保供养和残疾人保障工作。加强殡葬管理,开工建设凤凰山公墓和烈士陵园。建立健全社会养老服务体系,促进老年服务产业发展。

扎实实施民生工程。在全面完成省下达的“十项民生工程”、19件民生实事任务基础上,办好13项市定民生工程,切实解决事关群众切身利益的就业、安居、教育、医疗卫生、生态环境和基础设施建设等问题。继续抓好扶贫开发和移民后期扶持工作,实现8.13万人脱贫。

十、努力建设生态文明的美好家园

把生态文明建设放在更加突出位置,推动绿色发展、循环发展、低碳发展,加快资源节约型、环境友好型社会建设。

加大生态建设力度。巩固国家森林城市创建成果,大力开展植树造林,加强天然林保护和退耕还林工程建设,推进石漠化、漏水区治理。开展省级生态县、生态乡镇、生态村和生态工业园区建设。加强与南充、重庆等毗邻地区的合作,推进嘉陵江、渠江流域生态治理,共筑长江上游生态屏障。

集约节约利用资源。严守耕地保护红线,落实最严格的节约集约用地制度。启动创建国家循环经济示范城市,推进广安经开区循环化改造,积极推广农业循环经济发展模式。强化节能降耗工作,严格限制新上高耗能、高污染项目,加强重点领域、重点行业、重点企业节能。

加强环境保护。积极创建国家环保模范城市,强力推进主要污染物总量减排工作,加强农村面源污染治理,推进城镇污水、垃圾处理,实施全民水库及西溪河流域生态环境综合治理,抓好乡镇饮用水源保护。加强工业企业、城市扬尘污染治理,加快机动车尾气检测中心、PM2.5空气自动监测站建设。加强重点排污企业、重点流域断面在线监测监控,依法查处环境违法行为。

十一、切实维护社会和谐稳定

着眼于维护广大人民群众的根本利益,创新社会治理方式,提高社会治理水平,确保社会安定有序、人民安居乐业。

创新社会治理体制机制。坚持系统治理、依法治理、综合治理、源头治理,进一步改进社会治理方式。推进城乡社区建设,健全基层综合服务管理平台。扩大社会参与,加大社会组织培育引导力度,支持企事业单位、人民团体在社会治理中发挥更大作用。创新有效预防和化解社会矛盾体制,推行重大决策社会稳定风险评估,推动“大调解”体系向纵深延伸。探索建立专群结合社区矫正工作机制,打造“阳光信访”平台,推行网上受理信访制度,开展领导干部接访下访,畅通和规范群众诉求表达渠道。

扎实抓好安全生产工作。进一步健全安全生产责任体系,建成市县两级综合监管平台,夯实安全生产基层基础。突出抓好重点领域隐患排查和专项整治,扎实开展道路交通安全综合整治攻坚年行动,强化煤矿关闭重组和复工复产期间的安全监管,切实把各项要求落实到操作层面,推动安全生产形势持续好转。

深入推进平安广安建设。全面推进依法治市,深入实施“六五”普法,努力提高全社会法律意识。扎实开展法律服务和法律援助。深入推进城乡网格化服务管理,全面加强“五大民生警务”建设,大力实施社会治安突出问题专项整治行动,依法严厉打击刑事犯罪活动。完善、创新流动人口和特殊人群的服务管理。切实加强网络安全监管。推进基层应急管理规范化建设。加强食品药品监管体系、制度和能力建设,保障群众“舌尖上的安全”。整顿规范市场秩序,维护消费者合法权益,营造放心消费、安全消费的良好环境。加强国防动员和后备力量建设,不断巩固发展军政军民团结局面。进一步落实优抚安置政策。认真落实民族宗教政策。加强保密、人民防空、防震减灾等工作。做好邓小平同志诞辰110周年纪念活动安全保卫工作。

关于政府自身建设

抓好政府自身建设和改革,是做好政府工作的基础。我们将顺应全面深化改革和转型发展的新要求,围绕建设人民满意政府目标,扎实开展党的群众路线教育实践活动,以改革创新精神,全面加强政府自身建设。

加快转变政府职能。进一步简政放权,规范审批自由裁量权,充分发挥市场在资源配置中的决定性作用,更好发挥政府作用,努力为市场主体创造更加公平有序的发展环境。整合政务服务与公共资源交易平台建设,大力推进并联审批和网上审批,建立完善重点投资项目审批服务代办机制,切实提高行政效率。进一步加强公共服务、市场监管、社会管理、环境保护等职责,增加公共服务供给,提高公共服务水平,推行政府购买公共服务,促进城乡基本公共服务均等化。

深入推进依法行政。坚持以法治思维和法治方式履行职能,实施依法行政评估考核检查,把依法行政各项要求落实到政府工作的各个环节。深化政务信息公开,依托“网络理政”平台,大力推进政民互动。深化行政复议规范化建设,进一步加强行政调解工作。自觉接受人大及其常委会的监督,主动接受政协的民主监督,认真办理人大代表议案、建议、批评意见和政协委员提案,认真听取各民主党派、工商联、无党派人士和人民团体的意见,重视司法监督和舆论、公众监督。支持工会、共青团、妇联等群团组织开展工作。

努力改进工作作风。认真贯彻执行中省市改进作风各项规定,深入整治形式主义、官僚主义、享乐主义和奢靡之风。大力弘扬求真务实作风,切实改变文风会风,深入开展“走基层、解难题、办实事、惠民生”活动,积极为基层群众排忧解难。厉行节约、反对浪费,严控“三公”经费支出,不以任何形式和理由新建楼堂馆所。

切实抓好廉政建设。全面落实党风廉政建设各项部署,严格履行“一岗双责”,认真落实预防、监督和惩治制度,坚持用制度管权管事管人。大力推行行政权力清单制度,加大政府信息公开力度,建设电子政务大厅和电子监察平台。开展政风行风满意度测评,注重测评结果运用;加强廉政风险防控,加大工程建设、政府采购、民生资金等重点领域监督力度,及时解决损害群众利益的突出问题;强化财政、审计监督和行政监察,支持执纪执法机关查办案件,努力建设廉洁政府。

各位代表,我们正处在改革创新与感恩奋进的时代。改革释放红利,创新引领发展,追赶实现跨越。让我们在省委、省政府和市委的坚强领导下,抢抓机遇、乘势而进,攻坚克难、奋发图强,加快推进全市经济社会又好又快发展,为迈入全省发展第一方阵、全面建成小康社会而努力奋斗!
